# Biochemical Abnormalities Associated With Hypertension in Patients With Chronic Hepatitis B at Laquintinie Hospital, Cameroon: A Comparative Cross-Sectional Study

**DOI:** 10.7759/cureus.105096

**Published:** 2026-03-12

**Authors:** Stéphanie Larissa Nossupuwo, Jean-De-Dieu Tamokou

**Affiliations:** 1 Department of Biochemistry, University of Dschang, Dschang, CMR

**Keywords:** chronic hepatitis b, dyslipidemia, hypertension, liver dysfunction, metabolic abnormalities, renal impairment

## Abstract

Background

Hepatitis B virus (HBV) infection and hypertension (HTN) are major public health concerns worldwide. Both conditions independently contribute to hepatic, renal, and metabolic disturbances. However, the combined impact of HTN on biochemical abnormalities in chronic HBV patients remains insufficiently characterized. The objective of the present work was to assess the biochemical profile of patients with HTN and/or chronic HBV infection at Laquintinie Hospital in Douala, Cameroon, and to determine the impact of HTN and its severity on hepatic, renal, and metabolic markers in HBV carriers.

Methodology

A comparative cross-sectional design was carried out in the hepato-gastroenterology department of Laquintinie Hospital from December 2022 to January 2024, involving 401 individuals. Following detection of the hepatitis B surface antigen (HBsAg) and measurement of blood pressure, the participants were separated into four groups, including 92 hypertensive patients with HBV infection (HTN+/HBV+), 73 hypertensive patients without HBV (HTN+/HBV-), 96 normotensive HBV-infected patients (HTN-/HBV+), and 140 healthy controls (HTN-/HBV). Hepatic, renal, and cardiac biochemical markers were assayed using enzymatic and colorimetric methods from commercial kits and automated biochemical analyzers. Frequencies of biochemical abnormalities were compared across groups. Logistic regression analysis was performed to evaluate associations between HTN and biochemical abnormalities in HBV-positive and HBV-negative patients. The relationship between HTN grade and biochemical disturbances in HBV carriers was also examined.

Results

Patients with both HTN and HBV infection (HTN+/HBV+) exhibited the most pronounced biochemical alterations, including significantly elevated liver enzymes, bilirubin, cystatin C, albuminuria, triglycerides, LDL cholesterol, and fasting glucose, along with reduced glomerular filtration rate (GFR) and potassium levels (p < 0.01). The healthy group (HTN- / HBV-) showed the most favorable biochemical profiles. In HBV-positive patients, HTN was strongly and positively associated (odds ratio (OR) > 1; p < 0.01) with elevated alanine aminotransferase (ALT; OR = 12.00), aspartate aminotransferase (AST; OR = 41.94), total bilirubin (OR = 33.81), cystatin C (OR = 39.00), renal insufficiency (OR = 36.93), albuminuria (OR = 14.00), hypertriglyceridemia (OR = 7.36), and elevated LDL cholesterol (OR = 8.84). Similar renal and metabolic associations were observed in HBV-negative hypertensive patients, though liver-specific markers were less affected. Increasing HTN grade showed limited additional impact on most biochemical parameters, with significant differences observed only for total bilirubin, total cholesterol, and potassium (p < 0.05).

Conclusions

The coexistence of HTN and chronic HBV infection is associated with significant hepatic, renal, and metabolic disturbances. While the presence of HTN markedly exacerbates biochemical abnormalities in HBV patients, increasing HTN severity confers limited additional impact. Integrated management strategies targeting both blood pressure control and viral disease are essential to reduce organ-related complications in this high-risk population.

## Introduction

Hepatitis B virus (HBV) is a major viral infection and a major global public health issue that impacts millions of individuals worldwide and potentially progresses to chronic infection [1]. Chronicity exposes patients to a high risk of severe hepatic complications, such as cirrhosis and hepatocellular carcinoma, as well as extrahepatic manifestations [2]. These complications may impair the functions of several organs, including the liver, kidneys, and heart. Studies, sometimes contradictory, have suggested that patients with hepatitis B may have increased susceptibility to hypertension (HTN) due to chronic inflammation, insulin resistance, or the vascular impact of the virus [3,4]. HTN is a common and multifactorial cardiovascular disease whose diagnosis and management are complicated in patients with chronic liver diseases [5]. The increase in blood pressure observed during chronic hepatitis B may be life-threatening if not diagnosed and managed. However, it is difficult to identify vulnerable patients who may develop HTN, as the relationship between HTN and HBV is complex. Conversely, uncontrolled HTN may worsen liver damage or accelerate metabolic complications. However, in the Cameroonian context, there is limited data on the frequency of this association, the contributing factors, and its biochemical impact. The coexistence of chronic HBV and HTN raises questions about underlying pathophysiological mechanisms and biochemical abnormalities. On the one hand, during the progression of the disease, immune mechanisms involving viral antigens and specific anti-HBV antibodies are believed to contribute to various extrahepatic manifestations, including HBV-related glomerulonephritis, which may induce renal damage; damaged kidneys can no longer fully perform their regulatory functions on blood pressure [4,6]. On the other hand, as with the hepatitis C virus, HBV is thought to cause chronic liver damage through dyslipidemia [4]. In this context, identifying specific biochemical abnormalities associated with HTN in chronic HBV carriers is essential both for a deeper understanding of the pathophysiology of this comorbidity and for developing better management strategies. The coexistence of these two conditions may have a synergistic impact on patients’ clinical status and biological functions, particularly hepatic, renal, and metabolic ones. The objective of the present study was to evaluate the biochemical profile of patients with HTN and/or chronic HBV infection attending Laquintinie Hospital in Douala, Cameroon. The primary objective was to compare hepatic, renal, and metabolic biochemical markers across patient groups defined by the presence of HTN, chronic HBV infection, or both conditions. The secondary objective was to explore the association between HTN severity (by grade) and variations in these biochemical markers among HBV carriers. This analysis was conducted within an exploratory framework. 

## Materials and methods

Design and study population

This study was a cross-sectional analytical study conducted in the hepato-gastroenterology department of Laquintinie Hospital from December 2022 to January 2024. The study protocol received approval from the Institutional Ethics Committee for Human Health Research at the University of Douala, Douala, Cameroon (Ref: 3449 CEI-UDo/10/2022/T), along with authorization from Laquintinie Hospital. Participants were voluntary patients attending consultations at Laquintinie Hospital. The study was conducted in the hepato-gastroenterology department, the serology laboratory, and the biochemistry laboratory of the hospital from December 2022 to January 2024. The participants were healthy individuals and those suffering from HBV infection and HTN. Male and female participants of all ages were enrolled in the study. Eligible individuals included those with or without symptoms of the disease who provided written informed consent, with assent obtained for participants under 21 years of age. Exclusion criteria comprised a history of liver, kidney, or heart disease; hematological disorders; immunocompromised status; active alcohol consumption; pregnancy; positive serology for other viral hepatitis (C or D) or HIV co-infection; malaria; recent use of potentially hepatotoxic drugs; or any medication likely to influence biochemical parameters. Healthy control participants underwent baseline clinical screening, including medical history assessment and physical examination. Individuals with previously diagnosed diabetes mellitus, dyslipidemia, obesity (BMI ≥ 30 kg/m²), renal disease, or other chronic metabolic conditions were excluded from the control group. In addition, fasting glucose and lipid profiles were measured as part of the study protocol to ensure the absence of overt metabolic disorders. A total of 412 participants initially met the eligibility criteria. However, 11 individuals were excluded due to hemolyzed serum samples or incomplete data, resulting in a final sample size of 401 participants. Blood and urine samples were collected from all participants who provided informed consent. Detection of hepatitis B surface antigen (HBsAg) and measurement of blood pressure were used to classify participants as diseased or healthy and, subsequently, to divide them into four groups with comparable mean ages (p > 0.05): 140 negative participants, 73 hypertensive patients, 96 hepatitis B positive patients, and 92 hypertensive patients with hepatitis B.

Sample collection and preparation

Blood samples were collected from the cubital vein. Ten milliliters (10 ml) of blood were drawn from each patient by a trained laboratory technician under strict aseptic conditions. The blood was collected in a dry tube for hepatitis serology analysis. Samples were centrifuged at 3,000 rpm for 10 minutes to separate the serum, which was then sent to the serology laboratory for analysis. Urine samples were collected in sterile containers and sent to the biochemistry laboratory for analysis.

Chronic hepatitis B diagnosis

The diagnosis of hepatitis B was done by detecting the HBsAg antigen using the sandwich enzyme-linked immunosorbent assay (ELISA) method with the commercial kit (Fortress HBsAg, Fortress Diagnostics, Antrim, Northern Ireland, United Kingdom). A chronic HBV carrier was confirmed primarily through documented prior laboratory records indicating persistent HBsAg positivity for at least six months.

HTN diagnosis

Blood pressure was measured using a sphygmomanometer. Patients were placed at rest for 15 minutes in a seated position before measurement. Blood pressure was measured three times at five-minute intervals, and the mean was calculated. This measure was repeated for three days, and the diagnosis of HTN was confirmed over three months [7]. Values were recorded, compared to reference ranges, and patients were categorized following the guidelines of the National Committee for the Prevention, Detection, and Treatment of High Blood Pressure [7]. Reference values considered were normal blood pressure: < 120/80 mmHg; pre-HTN: 120-139/80-89 mmHg; stage 1 HTN: 140-159/90-99 mmHg; stage 2 HTN: 160-179/100-109 mmHg; and stage 3 HTN: ≥ 180/110 mmHg.

Evaluation of biochemical parameters

Biochemical analyses such as alanine aminotransferase (ALT), aspartate aminotransferase (AST), total bilirubin, and albumin were run on the CHINCAN EMP 168 semi-automatic biochemistry analyzer (Hangzhou Chincan Trading Co., Ltd., Hangzhou, China) using standard methods from commercial analyzer kits (BIOLABO, Maizy, France). Triglyceride, high-density lipoprotein (HDL) cholesterol, low-density lipoprotein (LDL) cholesterol, total cholesterol, and fasting glucose were run on the CHINCAN EMP 168 semi-automatic biochemistry analyzer using standard methods from commercial analyzer kits (LAB-CARE Diagnostics, Valsad, India). Cystatin C was run on an automatic biochemistry analyzer, MISPA CXL Pro (Agappe Diagnostics Ltd., Pattimattom, India), using standard methods from commercial analyzer kits (DiAgam, Liège, Belgium). Potassium was measured on the CHINCAN EMP 168 semi-automatic biochemistry analyzer using standard methods from commercial analyzer kits (potassium kit; Biosentec, Toulouse, France). Finally, urinary protein detection was performed on the URIT-50 urine analyzer (URIT Medical Electronic Co., Ltd., Guilin, China) using standard methods from commercial analyzer kits (LAB-CARE, India).

Glomerular filtration rate (GFR) was calculated using Hoek’s formula: [GFR (mL/min/1.73 m²) = -4.32 + 80.35 / cystatin C]. Normal renal function: < 0.85 mg/L (GFR > 90 mL/min/1.73 m²); mild renal impairment: 0.86 - 1.25 mg/L (GFR 60 - 89 mL/min/1.73 m²); moderate chronic renal impairment: 1.26 - 2.34 mg/L (GFR 30 - 59 mL/min/1.73 m²); severe chronic renal impairment: 2.35 - 4.16 mg/L (GFR 15 - 29 mL/min/1.73 m²); and end-stage renal disease: > 4.16 mg/L (GFR < 15 mL/min/1.73 m²) [8].

Statistical data analysis

Data collected were entered and coded into a Microsoft Excel database (Microsoft Corp., Redmond, WA, USA) and then analyzed using IBM SPSS Statistics software, version 26.0 (IBM Corp., Armonk, NY, USA). After cleaning the database and graphically verifying the normality of variable distributions, quantitative variables were summarized as means with standard deviations. Mean values of each biochemical parameter across the different groups were compared using the Waller-Duncan test when ANOVA indicated significant differences. The Waller-Duncan post hoc test was selected because it balances Type I and Type II error risks and performs well in datasets with unequal sample sizes across comparison groups. The Waller-Duncan conformity test was also applied to compare group means with reference values. Cut-off values provided by the assay kits were used to categorize parameters as normal (within the reference range) or abnormal (outside the reference range). The chi-square test was employed to compare the frequencies of biochemical abnormalities among the four groups. Multivariate logistic regression analysis was performed to evaluate associations between HTN and biochemical abnormalities in HBV-positive and HBV-negative patients. The multivariate logistic regression models included the following covariates: age, sex, and BMI. These variables were selected based on their known associations with metabolic and renal parameters. A p-value less than 0.05 was regarded as statistically significant.

## Results

Mean values of biochemical parameters in the different study groups

Patients with both HTN and HBV infection (HTN+/HBV+) showed the most pronounced biochemical alterations, including elevated ALT, AST, total bilirubin, cystatin C, serum albumin, albuminuria, triglycerides, total cholesterol, LDL cholesterol, and fasting glucose, alongside reduced GFR and potassium levels (Table 1). Groups without both conditions (HTN-/HBV-) had the most favorable profiles, with values mostly within reference ranges. HDL cholesterol remained similar across all groups.

**Table 1:** Mean values of biochemical parameters in the different study groups of participants Continuous variables were expressed as mean ± standard deviation; on the same line, values affected by the different superscript letters (a, b, c) are significantly different at a p-value < 0.05 (Waller-Duncan’s test). n : number of subjects per group; HTN+/HBV+: hypertension positive/hepatitis B positive; HTN-/HBV+: hypertension negative/hepatitis B positive; HTN+/HBV-: hypertension positive/hepatitis B negative; HTN-/HBV-: hypertension negative/hepatitis B negative; ALT: alanine aminotransferase; AST: aspartate aminotransferase; HDL: high-density lipoprotein; LDL: low-density lipoprotein; GFR: glomerular filtration rate

Biochemical parameters (reference values)	HTN^+^/HBV^+^ n = 92	HTN^-^/HBV^+^ n = 96	HTN^+^/HBV^-^ n = 73	HTN^-^/HBV^-^ n = 140
ALT (10 – 40 U/L)	59.23 ± 34.32ᵃ	47.69 ± 11.30ᵇ	29.67 ± 7.11ᶜ	29.27 ± 4.64ᶜ
AST (8 – 20 U/L)	52.07 ± 20.12ᵃ	41.48 ± 11.75ᵇ	24.46 ± 24.97ᶜ	19.43 ± 4.77ᶜ
Total bilirubin (< 12 mg/L)	10.29 ± 2.53ᵃ	8.89 ± 2.33ᵇ	8.49 ± 2.07ᵇ	7.17 ± 1.99ᶜ
Cystatin C (< 0.85 mg/L)	0.82 ± 0.22ᵃ	0.61 ± 0.18ᵇ	0.63 ± 0.10ᵇ	0.52 ± 0.11ᶜ
GFR (> 90 ml/min/1.73 m²)	97.81 ± 21ᵃ	131.44 ± 30.19ᵇ	124.65 ± 24.89ᵇ	152.21± 35.05ᶜ
Serum albumin (34 – 48 g/L)	40.20 ± 6.46ᵃ	36.54 ± 5.99ᵇ	34.86 ± 4.64ᵇ,ᶜ	33.60 ± 3.89ᶜ
Albuminuria (0 mg/dL)	13.35 ± 18.21ᵃ	1.75 ± 8.12ᵇ	0.55 ± 3.58ᵇ	0.00 ± 0.00ᵇ
Triglycerides (< 1.50 g/L)	3.70 ± 1.24ᵃ	2.75 ± 0.89ᵇ	3.54 ± 1.33ᵃ	2.49 ± 1.07ᵇ
Total cholesterol (< 2 g/L)	3.22 ± 1.16ᵃ	2.44 ± 0.75ᵇ	3.12 ± 1.30ᵃ	2.28 ± 0.85ᵇ
HDL cholesterol (> 0.45 g/L)	1.16 ± 0.16ᵃ	1.20 ± 0.18ᵃ	1.14 ± 0.18ᵃ	1.22 ± 0.27ᵃ
LDL cholesterol (< 1.31 g/L)	3.25 ± 0.85ᵃ	2.46 ± 0.63ᵇ	3.07 ± 1.04ᵃ	2.39 ± 0.66ᵇ
Blood glucose (0.60 – 1.26 g/L)	0.97 ± 0.22ᵃ	0.78 ± 0.14ᵇ	1.01 ± 0.28ᵃ	0.77 ± 0.09ᵇ
Potassium (3.50 – 5.50 mmol/L)	2.28 ± 0.69ᵃ	4.18 ± 0.76^c^	3.28 ± 1.13ᵇ	4.19 ± 0.75ᶜ

Frequencies of biochemical abnormalities in the different groups of the study population

The frequency of biochemical abnormalities was highest in patients with both HTN and HBV infection (HTN+/HBV+), who showed elevated liver enzymes, bilirubin, cystatin C, albuminuria, triglycerides, total cholesterol, LDL cholesterol, fasting glucose, and hypokalemia (Table 2). In contrast, the healthy group (HTN-/HBV-) exhibited the lowest prevalence of abnormalities, with most values within reference ranges. Lipid and glucose disturbances were more common in hypertensive groups, while HDL cholesterol remained similar across all groups. These findings indicate that coexisting HTN and HBV infection exacerbates liver, kidney, and metabolic dysfunctions.

**Table 2:** Frequencies of biochemical abnormalities in the different groups of the study population The chi-square test was employed to compare the frequencies of biochemical abnormalities among the four groups; a p-value less than 0.05 was regarded as statistically significant n: number of subjects per group; HTN+/HBV+: hypertension positive/hepatitis B positive; HTN-/HBV+: hypertension negative/hepatitis B positive; HTN+/HBV-: hypertension positive/hepatitis B negative; HTN-/HBV-: hypertension negative/hepatitis B negative; HDL: high-density lipoprotein; LDL: low-density lipoprotein

Biochemical parameters (reference values)	HTN^+/^HBV^+^ n (%) n = 92	HTN^+^/HBV^-^ n (%) n = 73	HTN^-^/HBV^+^ n (%) n = 96	HTN^-^/HBV^-^ n (%) n = 140	Chi-square (p-value)
Alanine aminotransferase					
Normal (10 – 40 IU/L)	11 (11.95)	69 (94.52)	22 (22.91)	140 (100)	273.85 (0.001)
High (> 40 IU/L)	81 (88.05)	4 (5.48)	74 (77.08)	0 (00)	
Aspartate aminotransferase					
Normal (8 – 20 IU/L)	3 (3.26)	40 (54.79)	4 (4.16)	82 (58.57)	131.58 (0.002)
High (> 20 IU/L)	89 (96.74)	33 (45.20)	92 (95.83)	58 (41.42)	
Total bilirubin					
Normal (< 12 mg/L)	74 (80.43)	72 (98.63)	86 (89.58)	139 (99.28)	33.80 (0.003)
High (> 12 mg/L)	18 (19.56)	1 (1.37)	10 (10.41)	1 (0.71)	
Cystatin C					
Normal (< 0.85 mg/L)	60 (65.21)	72 (98.63)	91 (94.79)	140 (100)	90.96 (0.002)
High (> 0.85 mg/L)	32 (34.78)	1 (1.37)	5 (5.21)	0 (0.00)	
Glomerular filtration rate					
Normal (> 90 ml/min/1.73 m²)	64 (69.56)	72 (98.63)	91 (94.79)	140 (100)	77.40 (0.002)
Mild renal insufficiency (60 – 89 ml/min/1.73 m²)	23 (25.00)	1 (1.37)	3 (3.12)	0 (0.00)	
Moderate chronic renal insufficiency (30 – 59 ml/min/1.73 m²)	5 (5.43)	0 (0.00)	2 (2.08)	0 (0.00)	
Albuminemia					
Normal (34 – 48 g/L)	71 (77.17)	47 (64.38)	56 (58.33)	64 (45.71)	31.46 (0.003)
Low (< 34 g/L)	20 (21.73)	26 (35.61)	37 (38.54)	76 (54.28)	
High (> 48 g/L)	1 (1.08)	0 (0.00)	3 (3.12)	0 (0.00)	
Albuminuria					
Normal (0 mg/L)	41 (44.56)	65 (89.04)	87 (90.62)	140 (100)	131.09 (0.002)
High (> 0 mg/L)	51 (55.44)	8 (10.96)	9 (9.38)	0 (0.00)	
Blood glucose					
Normal (0.6 – 1.2 g/L)	82 (89.13)	64 (87.67)	93 (96.87)	138 (98.54)	28.23 (0.003)
Low (< 0.7 g/L)	0 (0.00)	0 (0.00)	2 (2.08)	2 (1.42)	
High (> 1.2 g/L)	10 (10.87)	9 (12.33)	1 (1.04)	0 (0.00)	
Triglycerides					
Normal (< 1.5 g/L)	11 (11.95)	8 (10.96)	26 (27.08)	70 (50.00)	55.01 (0.003)
High (> 1.5 g/L)	81 (88.05)	65 (89.04)	70 (72.91)	70 (50.00)	
HDL cholesterol					
Normal (> 0.45 g/L)	92 (100)	73 (100)	96 (100)	136 (97.14)	3.74 (0.29)
High (> 0.7 g/L)	0 (0.00)	0 (0.00)	0 (0.00)	2 (1.42)	
Low (< 0.45 g/L)	0 (0.00)	0 (0.00)	0 (0.00)	2 (1.42)	
LDL cholesterol					
Normal (< 1.31 g/L)	7 (7.60)	10 (13.69)	25 (26.04)	59 (42.14)	41.59 (0.003)
High (> 1.31 g/L)	85 (92.40)	63 (86.31)	71 (73.96)	81 (57.85)	
Total cholesterol					
Normal (< 2 g/L)	11 (11.95)	11 (15.06)	20 (20.83)	19 (13.57)	49.10 (0.003)
Low (< 1.5 g/L)	14 (15.21)	19 (26.02)	33 (34.37)	75 (53.57)	
High (> 2 g/L)	67 (72.82)	43 (58.90)	43 (44.79)	46 (32.85)	
Potassium (kaliemia)					
Normal (3.5 – 5.5 mmol/L)	2 (2.17)	5 (6.84)	76 (79.16)	120 (85.71)	250.82 (0.001)
Hypokalemia (< 3.5 mmol/L)	88 (95.65)	63 (86.31)	16 (16.66)	18 (12.85)	
Hyperkalemia (> 5.5 mmol/L)	2 (2.17)	5 (6.84)	4 (4.16)	2 (1.42)	

Biochemical abnormalities associated with HTN in chronic HBV patients

Table 3 presents the biochemical abnormalities associated with HTN among patients with chronic HBV infection. In HBV-positive patients, HTN (HTN+/HBV+) was strongly associated with elevated liver enzymes (ALT: odds ratio (OR) = 12, AST: OR = 41.9), high total bilirubin (OR = 33.8), increased cystatin C (OR = 39), renal insufficiency (mild renal insufficiency (MRI): OR = 36.9; moderate chronic renal insufficiency (MCRI): OR = 30.9), albuminuria (OR = 14), elevated triglycerides (OR = 7.36), and high LDL cholesterol (OR = 8.84), all with p < 0.01. Low albumin levels were inversely associated with HTN (OR = 0.23, p < 0.01). In HBV-negative patients with HTN (HTN+/HBV-), similar associations were observed for ALT, cystatin C, GFR, albuminuria, triglycerides, and LDL cholesterol, while AST and bilirubin were not significantly affected. Blood glucose and HDL cholesterol showed no significant associations across groups. Potassium abnormalities (hypo- and hyperkalemia) were also strongly associated with HTN in both HBV-positive and HBV-negative patients. Overall, HTN in chronic HBV infection is linked to pronounced hepatic, renal, and metabolic disturbances, highlighting the compounding impact of HTN on liver and kidney function in these patients.

**Table 3:** Biochemical abnormalities associated with hypertension in chronic HBV patients Multivariate logistic regression analysis was performed to evaluate associations between hypertension and biochemical abnormalities in HBV+ and HBV- patients; a p-value less than 0.05 was regarded as statistically significant; OR = 1: no association, the predictor does not affect the odds of the outcome; OR > 1: the predictor is a risk factor; OR < 1: the predictor is a protective factor. HTN +/ HBV+: positive hypertension/positive viral hepatitis B; HTN-/ HBV+: negative hypertension/positive viral hepatitis B; HTN+/ HBV-: positive hypertension/negative viral hepatitis B; n: number of subjects per group; HDL: high density lipoprotein; LDL: low density lipoprotein; OR: odds ratio; CI: confidence interval; MRI: mild renal insufficiency; MCRI: moderate chronic renal insufficiency

Biochemical parameters (reference values)	Variations	HTN^+^ / HBV^+^ n = 92		HTN^-^ / HBV^+^ n = 96		HTN^+^ / HBV^- ^ n = 73	
		OR (CI)	p-value	OR (CI)	p-value	OR (CI)	p-value
Alanine aminotransferase (10 – 40 IU/L)	Normal	Reference		Reference		Reference	
	High	12 (1.26 – 12.66)	0.001	5.78 (2.62 – 12.74)	0.001	9.97 (3.03 – 32)	0.001
Aspartate aminotransferase (8 – 20 IU/L)	Normal	Reference		Reference		Reference	
	High	41.94 (12.65 – 139.06)	0.001	32.51 (11.31 – 93.48)	0.001	1.16 (0.65 – 2.06)	0.59
Total bilirubin (< 12 mg/L)	Normal	Reference		Reference		Reference	
	High	33.81 (4.42 – 258.29)	0.001	16.16 (2.03 – 128.49)	0.001	1.93 (0.11 – 31.31)	0.64
Cystatin C (< 0.85 mg/L)	Normal	Reference		Reference		Reference	
	High	39 (3.12 – 386.21)	0.001	4.03 (1.49 – 10.95)	0.001	10.21 (1.35 – 76)	0.001
Glomerular filtration rate (> 90 ml/min/1.73 m²)	Normal	Reference		Reference		Reference	
	MRI	36.93 (3.71 – 369.36)	0.001	33.88 (9.75 – 117.17)	0.001	14.27 (1.87 – 108.71)	0.001
	MCRI	30.97 (3.11 – 309)	0.001	8.71 (1.63 – 46.47)	0.001	1.03 (0.00 – 10)	1
Albuminemia (34 – 48 g/L)	Normal	Reference		Reference		Reference	
	Low	0.23 (0.13 – 0.43)	0.001	0.55 (0.32 – 0.94)	0.03	0.46 (0.26 – 0.83)	0.01
	High	1.21 (0.00 – 12.80)	1	4.60 (0.00 – 46.17)	1	0.73 (0.73 – 0.73)	1
Albuminuria (0 mg/dl)	Normal	Reference		Reference		Reference	
	High	14 (1.46 – 16.56)	0.001	12 (5.47 – 27.12)	0.001	14.50 (6.25 – 33)	0.001
Blood glucose (0.60 – 1.26 g/L)	Normal	Reference		Reference		Reference	
	Low	2.38 (1.00 – 10.04)	0.94	1.48 (0.20 – 10.72)	0.61	1 (0 – 1.06)	0.57
	High	16 (0.00 – 163)	0.68	14.39 (0.00 – 143)	0.99	1.88 (0.00 – 18.01)	0.96
Triglycerides (< 1.5 g/L)	Normal	Reference		Reference		Reference	
	High	7.36 (3.61 – 15)	0.001	2.69 (1.54 – 4.70)	0.001	8.12 (3.63 – 18.18)	0.001
HDL cholesterol (> 0.45 g/L)	Normal	Reference		Reference		Reference	
	Low	4.21 (0.14 – 7.26)	0.84	2.14 (0.17 - 5.19)	0.72	2.71 (0.84 – 6.02)	0.99
	High	1.23 (0.66 – 3.08)	0.79	1.17 (0.21 – 2.99)	0.98	3.13 (0.91 – 5.06)	0.83
LDL cholesterol (< 1.31 g/L)	Normal	Reference		Reference		Reference	
	High	8.84 (3.81 – 20.49)	0.001	2.06 (1.17 – 3.64)	0.01	4.58 (2.17 – 9.68)	0.001
Total cholesterol (< 2 g/L)	Normal	Reference		Reference		Reference	
	Low	0.27 (0.10 – 0.69)	0.001	0.41 (0.19 – 0.88)	0.02	0.43 (0.17 – 1.07)	0.07
	High	2.30 (1.02 – 5.20)	0.04	0.88 (0.41 – 1.88)	0.75	1.61 (0.68 – 3.78)	0.27
Potassium (3.50 – 5.50 mmol/L)	Normal	Reference		Reference		Reference	
	Low	293.33 (66.33 – 1297.05)	0.001	1.40 (0.67 – 2.91)	0.36	84 (29.78 – 236.86)	0.001
	High	60 (5.40 – 666.11)	0.001	3.15 (0.56 – 17.66)	0.19	60 (9.26–388.51)	0.001

Frequencies of biochemical abnormalities according to grades of HTN in chronic HBV carriers

In chronic HBV carriers, biochemical abnormalities were present across all HTN grades, but most parameters did not significantly vary with increasing HTN severity (Table 4). Total bilirubin and total cholesterol showed modest differences across grades (p = 0.01 and 0.04, respectively), while liver enzymes, kidney markers, lipids, glucose, and potassium remained largely unchanged.

**Table 4:** Frequencies of biochemical abnormalities according to grades of hypertension in chronic HBV carriers The chi-square test was employed to compare the frequencies of biochemical abnormalities among the three groups; a p-value less than 0.05 was regarded as statistically significant. /: chi-square value not calculated; n: number of subjects per group; HDL: high‑density lipoprotein; LDL: low‑density lipoprotein

Biochemical parameters	Grade 1 n (%) n = 16	Grade 2 n (%) n = 50	Grade 3 n (%) n = 26	Chi‑square (p‑value)
Alanine aminotransferase				
Normal (10 – 40 IU/L)	2 (12.50)	44 (88.00)	14 (53.84)	0.00 (0.99)
High (> 40 IU/L)	14 (87.50)	6 (12.00)	23 (46.16)	
Aspartate aminotransferase				
Normal (8 – 20 IU/L)	0 (0.00)	3 (6.00)	0 (0.00)	2.60 (0.29)
High (> 20 IU/L)	16 (100)	47 (94.00)	26 (100)	
Total bilirubin				
Normal (< 12 mg/L)	9 (56.25)	41 (82.00)	24 (92.30)	8.35 (0.01)
High (> 12 mg/L)	7 (43.75)	9 (18.00)	2 (7.70)	
Cystatin C				
Normal (< 0.85 mg/L)	10 (62.50)	32 (64.00)	18 (69.23)	0.26 (0.87)
High (> 0.85 mg/L)	6 (37.50)	18 (36.00)	8 (30.76)	
Glomerular filtration rate				
Normal (>90 ml/min/1.73 m²)	12 (75.00)	34 (68.00)	18 (69.23)	1.00 (0.90)
Mild renal insufficiency (60 – 89 ml/min/1.73 m²)	3 (18.75)	14 (28.00)	6 (23.07)	
Moderate chronic renal insufficiency (30–59 ml/min/1.73 m²)	1 (6.25)	2 (4.00)	2 (7.69)	
Serum albumin				
Normal (34 – 48 g/L)	11 (68.75)	39 (78.00)	21 (80.76)	5.10 (0.27)
Low (< 34 g/L)	4 (25.00)	11 (22.00)	5 (19.23)	
High (> 48 g/L)	1 (6.25)	0 (0.00)	0 (0.00)	
Albuminuria				
Normal (0 mg/dl)	9 (56.25)	20 (40.00)	12 (46.15)	1.33 (0.51)
High (> 0 mg/dl)	7 (43.75)	30 (60.00)	14 (53.85)	
Blood glucose				
Normal (0.6–1.2 g/L)	13 (81.25)	45 (90.00)	24 (92.30)	1.33 (0.51)
High (> 1.2 g/L)	3 (18.75)	5 (10.00)	2 (7.69)	
Triglycerides				
Normal (< 1.5 g/L)	4 (25.00)	5 (10.00)	2 (7.69)	3.21 (0.20)
High (> 1.5 g/L)	12 (75.00)	45 (90.00)	24 (92.30)	
HDL cholesterol				
Normal (> 0.45 g/L)	16 (100)	50 (100)	26 (100)	/
Low (< 0.45 g/L)	0 (0.00)	0 (0.00)	0 (0.00)	
LDL cholesterol				
Normal (< 1.31 g/L)	2 (12.50)	4 (8.00)	1 (3.84)	1.07 (0.58)
High (> 1.31 g/L)	14 (87.50)	46 (92.00)	25 (96.16)	
Total cholesterol				
Normal (< 2 g/L)	5 (31.25)	6 (12.00)	1 (3.84)	10.03 (0.04)
Low (< 1.5 g/L)	4 (25.00)	5 (10.00)	4 (15.38)	
High (> 2 g/L)	7 (43.75)	39 (78.00)	21 (80.76)	
Serum potassium				
Normal (3.5–5.5 mmol/L)	1 (6.25)	1 (2.00)	0 (0.00)	4.58 (0.33)
Low (< 3.5 mmol/L)	14 (87.50)	49 (98.00)	25 (96.16)	
High (> 5.5 mmol/L)	1 (6.25)	0 (0.00)	1 (3.84)	

Association of biochemical parameters with grades of HTN in chronic HBV carriers

In chronic HBV carriers, most biochemical parameters were not significantly associated with HTN grade (Table 5). Exceptions included total bilirubin, total cholesterol, and potassium, which showed significant associations with higher HTN grades (p < 0.05). These findings suggest that while the presence of HTN affects biochemical disturbances, increasing HTN severity has a limited additional impact in chronic HBV patients.

**Table 5:** Association of biochemical parameters with grades of hypertension in chronic HBV carriers Multivariate logistic regression analysis was performed to evaluate associations between hypertension and biochemical abnormalities in HBV-positive and HBV-negative patients; a p-value less than 0.05 was regarded as statistically significant; OR = 1: no association, the predictor does not affect the odds of the outcome; OR > 1: the predictor is a risk factor; OR < 1: the predictor is a protective factor. OR: odds ratio; CI: confidence interval; MRI: mild renal insufficiency; MCRI: moderate chronic renal insufficiency; n: number of subjects per group; HBV: hepatitis B virus; LDL: low-density lipoprotein

Biochemical parameters (reference values)	Variations	Grade 3 n = 26		Grade 2 n = 50	
		OR (CI)	p-value	OR (CI)	p-value
Alanine aminotransferase (10 – 40 IU/L)	Normal	Reference		Reference	
	High	1.09 (0.16 – 7.38)	0.92	1.04 (0.19 – 5.79)	0.95
Aspartate aminotransferase (8 – 20 IU/L)	Normal	Reference		Reference	
	High	1 (0.00 – 10)	1	2.07 (2.07 – 6.91)	1
Total bilirubin (< 12 mg/L)	Normal	Reference		Reference	
	High	0.10 (0.01 – 0.61)	0.01	0.28 (0.08 – 0.95)	0.04
Cystatin C (< 0.85 mg/L)	Normal	Reference		Reference	
	High	0.74 (0.20 – 2.74)	0.65	0.93 (0.29 – 3)	0.91
Glomerular filtration rate (> 90 ml/min/1.73 m²)	Normal	Reference		Reference	
	MRI	1.33 (0.27 – 6.38)	0.71	1.64 (0.40 – 6.74)	0.48
	MCRI	1.33 (0.10 – 16.39)	0.82	0.70 (0.05 – 8.50)	0.78
Albuminemia (34 – 48 g/L)	Normal	Reference		Reference	
	Low	0.65 (0.14 – 2.94)	0.58	0.77 (0.20 – 2.92)	0.70
	High	3.69 (3.69 – 7.47)	0.91	3 (0.00 – 3.83)	0.96
Albuminuria (0 mg/dl)	Normal	Reference		Reference	
	High	1.50 (0.42 – 5.25)	0.52	1.92 (0.61 – 6.02)	0.25
Blood glucose (0.6 – 1.2 g/L)	Normal	Reference		Reference	
	High	0.36 (0.05 – 2.44)	0.29	0.48 (0.10 – 2.28)	0.35
Triglycerides (< 1.5 g/L)	Normal	Reference		Reference	
	High	4 (0.63 – 25.02)	0.13	3 (0.69 – 12.92)	0.14
LDL cholesterol (< 1.31 g/L)	Normal	Reference		Reference	
	High	3.57 (0.29 – 42.99)	0.31	1.64 (0.27 – 9.93)	0.58
Total cholesterol (< 2 g/L)	Normal	Reference		Reference	
	Low	5 (0.38 – 64.38)	0.21	1.04 (0.17 – 6.12)	0.96
	High	4.15 (1.48 – 15.12)	0.02	4.64 (1.10 – 19.47)	0.03
Potassium (3.5 – 5.5 mmol/L)	Normal	Reference		Reference	
	Low	29.3 (6.63 – 129.70)	0.001	3.50 (0.20 – 59.59)	0.38
	High	11.87 (1.26 –118.70)	1	1 (0.00 – 1.63)	0.99

## Discussion

The present findings demonstrate that the coexistence of HTN and chronic HBV infection exerts a synergistic and deleterious effect on hepatic, renal, and metabolic parameters. Patients with both conditions (HTN+/HBV+) consistently exhibited the most pronounced biochemical alterations, whereas individuals without HTN or HBV infection (HTN-/HBV-) maintained largely normal profiles. This pattern underscores the compounding pathophysiological burden imposed by dual disease states.

The marked elevation of liver enzymes (ALT and AST) and total bilirubin in the HTN+/HBV+ group suggests enhanced hepatocellular injury and impaired hepatic function. Chronic HBV infection alone induces persistent hepatic inflammation and immune-mediated hepatocyte damage [9]. When combined with HTN, which is associated with endothelial dysfunction, vascular remodeling, and impaired hepatic perfusion [10], liver injury may be amplified [11]. The particularly high ORs observed for AST and bilirubin reinforce the strength of this association and suggest that HTN may aggravate underlying hepatic inflammation or fibrosis in HBV-infected individuals. Renal impairment was also more pronounced in patients with both conditions, as evidenced by elevated cystatin C, reduced GFR, and increased albuminuria. HTN is a well-established driver of glomerular damage through increased intra-glomerular pressure and nephrosclerosis [12]. In chronic HBV infection, immune complex deposition and virus-associated glomerulopathies further compromise renal function [13]. The strong associations observed between HTN and markers of renal insufficiency in HBV-positive patients suggest additive or possibly synergistic mechanisms accelerating kidney dysfunction. Metabolic disturbances, including elevated triglycerides, total cholesterol, LDL cholesterol, and fasting glucose, were more frequent in hypertensive groups, particularly among HBV-positive patients. HTN commonly clusters with dyslipidemia and impaired glucose metabolism as components of cardiometabolic syndrome [14]. Chronic liver disease may further alter lipid handling and insulin sensitivity [15]. The persistence of normal HDL cholesterol levels across groups suggests selective lipid abnormalities rather than a generalized lipid disorder, which may reflect disease-specific metabolic pathways. 

Within the HBV-positive population, HTN was strongly associated with hepatic cytolysis, hyperbilirubinemia, renal dysfunction, albuminuria, and atherogenic lipid abnormalities. Interestingly, low serum albumin was inversely associated with HTN. This finding may reflect the complex interplay between hepatic synthetic function and plasma volume regulation. Lower albumin levels often indicate more advanced liver dysfunction [16], which may coexist with hemodynamic alterations that do not necessarily parallel systemic HTN patterns. In contrast, among HBV-negative hypertensive patients, liver-specific abnormalities such as AST elevation and hyperbilirubinemia were not significant. This distinction suggests that hepatic injury in hypertensive patients without HBV may be less severe or mediated by different mechanisms, such as nonalcoholic fatty liver changes rather than viral inflammation [15]. However, renal and metabolic disturbances remained strongly associated with HTN regardless of HBV status, confirming HTN's independent contribution to kidney and cardiometabolic risk [17]. The strong association of potassium abnormalities (both hypo- and hyperkalemia) with HTN in both HBV-positive and HBV-negative groups likely reflects the influence of antihypertensive therapies, renal impairment, or alterations in the renin-angiotensin-aldosterone system, as recognized in HTN management guidelines [18]. Electrolyte monitoring is therefore particularly important in these patients. 

Interestingly, although the presence of HTN significantly influenced biochemical abnormalities, increasing the HTN grade had a limited additional impact among chronic HBV carriers. Only total bilirubin, total cholesterol, and potassium levels varied significantly with HTN severity. Most liver enzymes, renal markers, and metabolic parameters did not show progressive deterioration across HTN grades. This finding suggests that the primary determinant of biochemical disruption may be the presence of HTN itself rather than its severity. Once vascular and hemodynamic alterations are established, additional increments in blood pressure may not proportionally intensify hepatic or renal injury in chronic HBV infection. 

Collectively, these findings highlight the relevance of integrated cardiovascular and hepatic management in chronic HBV patients. The coexistence of HTN was associated with a higher prevalence of liver dysfunction, renal impairment, and metabolic abnormalities. Early detection and aggressive management of HTN in HBV-infected individuals may help mitigate progressive organ damage. Routine monitoring of liver enzymes, renal function markers (including cystatin C and GFR), lipid profiles, glucose, and electrolytes should be emphasized in this population. A multidisciplinary approach addressing viral control, blood pressure regulation, and metabolic risk reduction is likely essential to improving long-term outcomes.

Limitations

Participants were recruited from a tertiary hospital setting, which may preferentially include individuals seeking medical care and may not fully represent asymptomatic HBV carriers in the general community. This limitation demonstrates that findings may not be fully generalizable to community-based HBV populations. The very high ORs (e.g., OR > 200 for potassium abnormalities) suggest potential sparse cell counts or quasi-complete separation in logistic regression models. The limitations regarding incomplete data on antihypertensive medications could influence renal and metabolic parameters. HBV viral load, hepatitis B 'e' antigen status, and liver fibrosis stage were not systematically available for all participants and were therefore not included in the analysis, although disease activity may affect biochemical parameters. Due to the cross-sectional design, temporality and causality cannot be established, and the findings should be interpreted as associations. Finally, the lack of graded association may reflect sample size limitations or the cross-sectional nature of the study.

## Conclusions

Overall, the coexistence of HTN and chronic HBV infection is associated with significant hepatic, renal, and metabolic disturbances. While HTN alone contributes to renal and metabolic abnormalities, its presence in HBV-infected patients markedly exacerbates liver injury and kidney dysfunction. However, increasing HTN severity appears to confer limited additional biochemical deterioration beyond its mere presence. These findings reinforce the concept of compounded organ burden in patients with dual chronic conditions and support the need for prospective studies to determine whether integrated management strategies can improve biochemical outcomes.
